# Improved image quality with deep learning reconstruction – a study on a semi-anthropomorphic upper-abdomen phantom

**DOI:** 10.1016/j.redii.2023.100022

**Published:** 2023-01-13

**Authors:** Tormund Njølstad, Anselm Schulz, Kristin Jensen, Hilde K. Andersen, Anne Catrine T. Martinsen

**Affiliations:** a Department of Radiology and Nuclear Medicine, Oslo University Hospital Ullevål, Oslo 0450, Norway; b Department of Radiology, Haukeland University Hospital, Bergen, Norway; c Department of Physics and Computational Radiology, Oslo University Hospital, Oslo, Norway; d Faculty of Health Sciences, Oslo Metropolitan University, Oslo, Norway; e Sunnaas Rehabilitation Hospital, Nesodden, Norway

**Keywords:** Abdominal CT, Deep learning reconstruction, Image quality

## Abstract

**Purpose:**

To assess image quality of a deep learning reconstruction (DLR) algorithm across dose levels using a semi-anthropomorphic upper-abdominal phantom, and compare with filtered back projection (FBP) and hybrid iterative reconstruction (IR).

**Material and methods:**

CT scans obtained at five dose levels (CTDI_vol_ 5, 10, 15, 20 and 25 mGy) were reconstructed with FBP, hybrid IR (IR50, IR70 and IR90) and DLR of low (DLL), medium (DLM) and high strength (DLH) in 0.625 mm and 2.5 mm slices. CT number, homogeneity, noise, contrast, contrast-to-noise ratio (CNR), noise texture deviation (NTD; a measure of IR-specific artifacts), noise power spectrum (NPS) and task-based transfer function (TTF) were compared between reconstruction algorithms.

**Results:**

CT numbers were highly consistent across reconstruction algorithms. Image noise was significantly reduced with higher levels of DLR. Noise texture (NPS and NTD) was with DLR maintained at comparable levels to FBP, contrary to increasing levels of hybrid IR. Images reconstructed with DLR of low and high strength in 0.625 mm slices showed similar noise characteristics to 2.5 mm slice FBP and IR50, respectively. Dose-reduction potential based on image noise with IR50 as reference was estimated to 35% for DLM and 74% for DLH.

**Conclusions:**

The novel DLR algorithm demonstrates robust noise reduction with maintained noise texture characteristics despite higher algorithm strength, and may have overcome important limitations of IR. There may be potential for dose reduction and additional benefit from thin-slice reconstruction.

## 1 Introduction

A rapid increase in the use of CT imaging has been observed over the last decades [1]. Driven by the increase in radiation exposure, a public health concern towards the increased risk for radiation-induced malignancy has emerged [2]. Thus, novel methods to reduce radiation dose delivered to the patient without compromising image quality are aspired in clinical practice.

Filtered back-projection (FBP) has been the historical standard for CT image reconstruction, later accompanied by iterative reconstruction (IR) algorithms [3], [4], [5]. Several generations of IR have emerged over the recent years, including hybrid IR and model-based IR, with demonstrated potential for dose reduction [6]. However, application of IR to reduce image noise also alters image texture [7]. Concerns have been raised that this may deteriorate image quality, primarily affecting low-contrast tasks when radiation dose is reduced below a certain threshold [8].

Driven by algorithm development and significant advances in computational power, deep learning in image processing has gained momentum over the last decades [9]. A reconstruction algorithm based on deep learning reconstruction (DLR) received clinical approval in 2019 (TrueFidelity, GE Healthcare, Waukesha, WI). Other vendor-specific algorithms for deep learning reconstruction are also developed. As explained by a technical white paper [10], the DLR algorithm has been trained with high-dose and low-dose FBP datasets across a large number of phantom and patient cases in order to learn how to suppress image noise without compromising image quality. The DLR algorithm strives to improve image quality in previously challenging areas, such as low-dose imaging and scanning of obese individuals.

Noteworthy, although novel noise reduction methods such as DLR may seem promising, it is imperative that they are comprehensively evaluated in phantom and clinical studies to ensure that established clinical benefits are not compromised in the pursuit for dose reduction [8]. Studies on standard phantoms using conventional slice thickness have shown that the DLR algorithm can reduce noise magnitude while maintaining noise texture and high-contrast spatial resolution [11, 12]. Clinical studies have demonstrated improved perceived overall image quality of images reconstructed with DLR, for both standard dose and reduced dose CT [13, 14]. Furthermore, clinical experience shows that there may be benefit of enhanced spatial resolution in images reconstructed in thinner slices than conventional thicker slices, although only explored on standard dose CT [15].

On this basis, this study set out to compare the image quality of CT images reconstructed with DLR in both 0.625 mm and 2.5 mm slice thickness with FBP and IR for a selection of quantitative image quality metrics using a customized semi-anthropomorphic upper-abdominal phantom.

## 2 Materials and methods

**Fig. 1:**
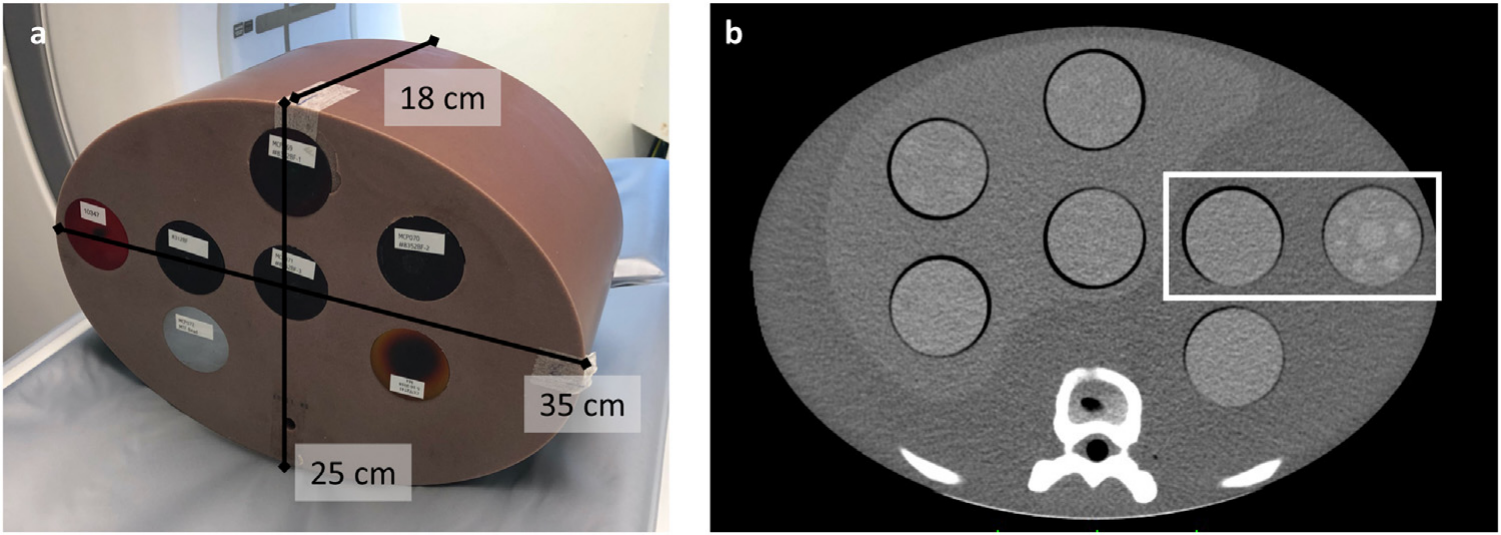
Photograph (a) and CT image (b) of semi-anthropomorphic upper-abdomen phantom applied in the study, containing a homogenous insert and a low-contrast insert for quantitative image quality analysis (white rectangle).

To best simulate a clinical setting, this study was conducted on a customized semi-anthropomorphic upper-abdomen phantom (Liver Phantom model CCT288, The Phantom Laboratory, Salem, NY), with dimensions 35 cm in the lateral, 25 cm in the anterior posterior, and 18 cm in the craniocaudal direction (Fig. 1a) containing seven cylindrical 5-cm wide inserts where four are located in the semi-anthropomorphic liver parenchyma. For this study, the phantom was equipped with a homogeneous insert and a low-contrast insert for quantitative image quality analyses (Fig. 1b). The homogeneous insert had a CT number of approximately 58 HU whereas the phantom background had a CT number of approximately 18 HU. The low-contrast insert contained a center lesion approximately 15 mm diameter, with a CT number of approximately 80 HU compared to a insert background of approximately 55 HU.

### 2.1 Image acquisition and reconstruction

The phantom was scanned in June 2019 using a 256-slice multidetector CT scanner (GE Revolution; GE Healthcare; Waukesha, WI). Scans were obtained at five radiation dose levels considered clinically relevant [16], with a CT dose index (CTDIvol) of 5, 10, 15, 20 and 25 mGy, achieved by applying a tube current of 75, 150, 225, 300 and 375 mAs, respectively. Scans were done in helical mode, with a constant tube voltage at 120 kV in line with our institutional protocol for abdominal CT, 80 mm detector collimation (128 × 0.625 mm) and pitch of 0.5. For each dose level, the phantom was scanned three separate times to increase data and reduce statistical variation for quantitative image quality analyses. Raw data were reconstructed applying seven different reconstruction algorithms; standard FBP, three levels of hybrid IR considered clinically relevant (ASiR-V 50% [IR50], ASiR-V 70% [IR70] and ASiR-V 90% [IR90]) and three strengths of DLR (TrueFidelity of low [DLL], medium [DLM] and high strength [DLH]) in 2.5 mm slice thickness. FBP was included as the DLR algorithm has been trained on FBP datasets. In addition, as there may be benefit from enhanced spatial resolution and better visualization of structures using out of plane reformation when reconstructing images in thinner slices, DLR images were also reconstructed with 0.625 mm slice thickness for comparison. For reconstruction of the DLR images, raw data were sent to the DLR vendor (GE Healthcare; Waukesha, WI) as part of a research agreement.

### 2.2 Image quality assessment

For each scan, CT images covering a 10 cm portion of the phantom were selected for quantitative analyses (i.e., 40 consecutive CT images for 2.5 mm slices and 160 CT images for 0.625 mm slices). Thus, a total of 11,400 CT images were used for quantitative analysis.

Quantitative image quality was assessed using a tailored script in the Matlab environment (Mathworks; Natick, MA). For each CT image, a region of interest (ROI) measuring 41 by 41 pixels (28.1 × 28.1 mm) was placed centrally in the homogenous insert, four smaller ROIs measuring 21 by 21 pixels (14.4 × 14.4 mm) were placed in the peripheral part of the homogenous insert and one ROI measuring 21 by 21 pixels (14.4 × 14.4 mm) was placed in the phantom background (Suppl. Fig. 1a).

For each ROI, mean and standard deviation in CT numbers were calculated, where the latter was regarded as a marker of image noise. Contrast-to-noise ratio (CNR) was assessed by comparing the phantom background to the homogenous insert, simulating a hypoattenuating lesion, and was computed by the following formula:

(1) $$CNR=\frac{\mu_{1}-\mu_{2}}{\sigma }$$

where μ denotes the signal (mean CT number), subscripts 1 and 2 represent the two target ROIs (homogenous insert and phantom background, respectively) and σ the standard deviation (noise) in the homogenous insert. Noise texture deviation (NTD), a metric reported in literature to correlate with IR-specific artifacts [7], was defined as the proportion of pixels within a ROI deviating more than three standard deviations from the mean, and calculated by the following formula:

(2) $$f_{3\sigma }=\frac{1}{n}\left\{i\left|p_{i}-\bar{p}\right|>3\sigma \right\}$$

where n denotes number of pixels within the ROI, $p_{i}$ the CT number in a given pixel, i, $\bar{p}$ the average CT number within the ROI, and σ the standard deviation in CT numbers. Homogeneity was defined as the maximum difference in CT numbers between the ROIs in the homogenous insert.

Noise power spectrum (NPS) and task-based transfer function (TTF) were measured using the imQuest open-source CT image analysis software package by Samei et al., available at https://deckard.duhs.duke.edu/∼samei/tg233.html [17]. NPS was assessed by placing four quadratic ROIs radially distributed within the homogenous insert across 40 slices for 2.5 mm slice thickness and 160 images for 0.625 mm slice thickness, generating 1D NPS profiles representing the radial average of the 2D NPS for each scan (Suppl. Fig. 1a). NPS curves used for comparison across reconstruction techniques were obtained by averaging the 1D NPS across the three scans with the same dose level, and normalized by dividing the NPS by the total noise power. The average spatial frequency, denoted f_avg_, was used as a scalar to compare noise texture between reconstruction techniques, and calculated by the following formula [18]:

(3) $$f_{avg}=\frac{\sum f\cdot NPS\left(f\right)}{\sum NPS\left(f\right)}$$

where f denotes the spatial frequency, and NPS(f) the 1D NPS.

TTF was assessed by placing a circular ROI around the centermost lesion in the low-contrast insert in the imQuest software (Suppl. Fig. 1c). As the low-contrast rod measured approximately 3 cm in the z-direction, ten consecutive image slices were obtained from each scan, and TTF curve averages were calculated. Care was taken to position the circular ROI so that it did not include the smaller surrounding low-contrast objects. Due to low contrast-to-noise ratio conditions, TTF measurements were only performed for the 25 mGy dose level. To compare low-contrast spatial resolution between reconstruction techniques, a scalar representing the spatial frequency in which TTF reached 50% was applied, denoted f_50%_.

### 2.3 Statistical analysis

Image noise, CNR and NTD averages were calculated for each reconstruction algorithm and compared using t-statistics at the 95% significance level. Estimation of dose reduction potential of DLR based on image noise was performed using linear regression with logarithmic transformation of image noise and CT dose index, which provided the best model fit. 95% confidence intervals for dose reduction estimates were calculated using the delta method [19]. Statistical analyses were performed using R statistical software (version 3.0.4, R Foundation for Statistical Computing, Vienna, Austria, https://www.r-project.org/).

## 3 Results

CT numbers were highly consistent for the investigated reconstruction algorithms, with mean CT number in the homogenous insert ranging 53.6–60.1 HU for FBP, 53.8–60.0 for IR, 54.1–59.9 HU for 2.5 mm DLR and 53.0–61.6 HU for 0.625 mm DLR. Difference in CT number between reconstruction algorithms ranged 0.07–1.64 HU. Homogeneity, representing the largest difference in CT number between the different ROIs in the homogenous insert, ranged 0.2–5.6 HU for FBP, 0.1–5.5 HU for IR, 0.1–4.6 HU for 2.5 mm DLR and 0.1–8.2 HU for 0.625 mm DLR.

**Fig. 2:**
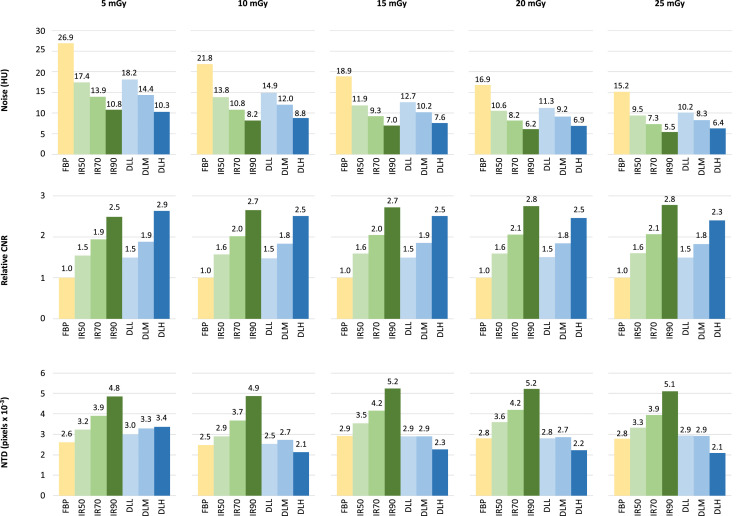
Bar plots of noise magnitude (top row), relative contrast-to-noise ratio (CNR, middle row) and noise texture deviation (NTD, bottom row) across dose levels for 2.5 mm images reconstructed with filtered back projection (FBP), three levels of hybrid iterative reconstruction (IR50, IR70 and IR90) and three strengths of deep learning reconstruction (low [DLL], medium [DLM] and high strength [DLH]). CNR-measurements are relative to FBP within the same dose level.

A comparison of noise, relative CNR and NTD for each reconstruction technique and dose level for 2.5 mm slices is presented in Fig. 2. Relative to FBP, image noise was on average reduced by 33% with DLL (range, 29–35%), 46% with DLM (range, 42–48%) and 60% with DLH (range, 56–63%);(all *p*<.001 for difference). Compared to FBP, relative CNR was on average 1.49 times higher for DLL (range 1.39–1.56), 1.85 times higher for DLM (range, 1.71–1.98) and 2.5 times higher for DLH (range, 2.26–2.83);(all *p*<.001 for difference). NTD increased with higher levels of IR, with mean NTD of 2.7 × 10^−3^ for FBP, 3.3 × 10^−3^ for IR50, 4.0 × 10^−3^ for IR70 and 5.1 × 10^−3^ for IR90. With DLR, NTD roughly paralleled that of FBP for DLL and DLM with mean NTD of 2.8 and 2.9 × 10^−3^, respectively, and was slightly lower for DLH with mean NTD of 2.4 × 10^−3^. With the image noise of IR50 as reference, the potential for dose reduction while maintaining a comparable level of image noise was estimated to 8.6 mGy for DLM (95% CI 8.4–8.8 mGy, *p*<.001) and 18.4 mGy for DLH (95% CI 18.3–18.6 mGy, *p*<.001), equivalent to a dose reduction of 35% for DLM and 74% for DLH with the 25 mGy dose level as reference.

**Fig. 3:**
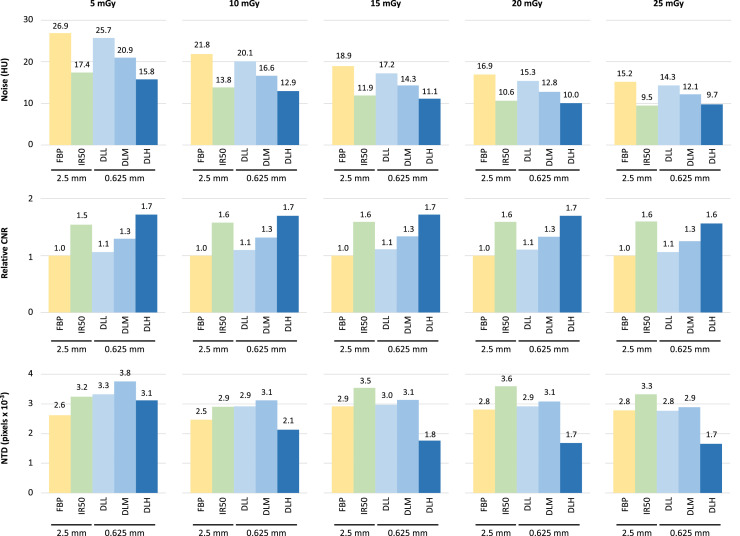
Bar plots of noise magnitude (top row), relative contrast-to-noise ratio (CNR, middle row) and noise texture deviation (NTD, bottom row) measurements across dose level for 2.5 mm images reconstructed with filtered back projection (FBP) and iterative reconstruction (IR50) and 0.625 mm images reconstructed with three strengths of deep learning reconstruction (low [DLL], medium [DLM] and high strength [DLH]). CNR measurements are relative to 2.5 mm FBP.

An overview of image noise, relative CNR and NTD for DLR images reconstructed in 0.625 mm slices compared to 2.5 mm FBP and 2.5 mm IR50 is presented in Fig. 3. For noise and relative CNR measurements, 0.625 mm DLL roughly paralleled that of 2.5 mm FBP whereas 0.625 mm DLH roughly paralleled that of 2.5 mm IR50. NTD was roughly equivalent to 2.5 mm FBP for 0.625 mm DLR for the higher dose levels (15–25 mGy), whereas DLR paralleled 2.5 mm IR50 for the lower dose levels (5 and 10 mGy).

**Fig. 4:**
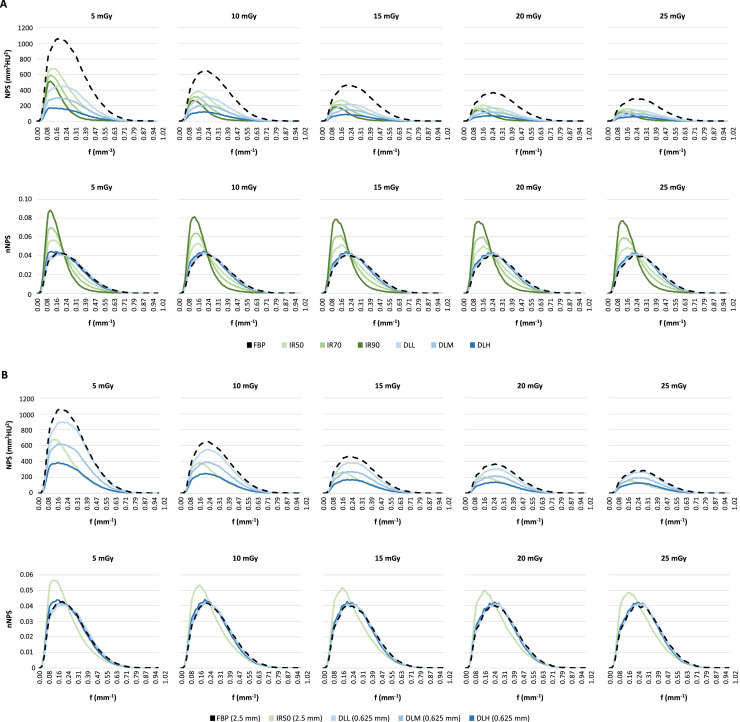
Plots of radially averaged noise power spectrum (NPS; top row) and normalized noise power spectrum (nNPS; bottom row) for each dose level and reconstruction algorithm. Images reconstructed with filtered back projection (FBP), three levels of hybrid iterative reconstruction (IR50, IR70 and IR90) and three strengths of deep learning reconstruction (low [DLL], medium [DLM] and high strength [DLH]) with 2.5 mm slice thickness (A) and 0.625 mm slice thickness (B).

NPS and normalized NPS (nNPS) curves for each reconstruction algorithm and dose level are presented in Fig. 4. NPS measurements showed substantially different noise texture with increasing levels of hybrid IR, with 14–16% lower f_avg_ for IR50, 24–25% lower for IR70 and 37–38% lower for IR90, depending on dose level. For images reconstructed with DLR, the noise texture was comparable to FBP, with a difference in f_avg_ of 1–2% for DLL, 1–4% for DLM and 6% for DLH, depending on dose level (Table 1).

**Table 1:** Average spatial frequencies (NPS f_avg_) for noise power spectrum curves across dose levels and reconstruction algorithms, and difference from FBP.

Dose level	5 mGy		10 mGy		15 mGy		20 mGy		25 mGy	
Reconstruction algorithm	NPS f_avg_ (mm^−1^)	%	NPS f_avg_ (mm^−1^)	%	NPS f_avg_ (mm^−1^)	%	NPS f_avg_ (mm^−1^)	%	NPS f_avg_ (mm^−1^)	%
FBP	0.28	–	0.30	–	0.30	–	0.31	–	0.31	–
IR50	0.24	−16%	0.25	−15%	0.26	−15%	0.26	−14%	0.27	−14%
IR70	0.21	−25%	0.22	−25%	0.23	−25%	0.23	−24%	0.23	−24%
IR90	0.18	−37%	0.19	−37%	0.19	−38%	0.19	−37%	0.19	−37%
DLL	0.29	1%	0.29	−1%	0.30	−2%	0.30	−2%	0.30	−2%
DLM	0.28	−1%	0.29	−3%	0.29	−3%	0.30	−4%	0.30	−4%
DLH	0.27	−6%	0.28	−6%	0.29	−6%	0.29	−6%	0.29	−6%

Images reconstructed with filtered back projection (FBP), three strengths of hybrid iterative reconstruction (IR50, IR70 and IR90) and three strengths of deep learning reconstruction (low [DLL], medium [DLM] and high strength [DLH]) in 2.5 mm slice thickness.

**Fig. 5:**
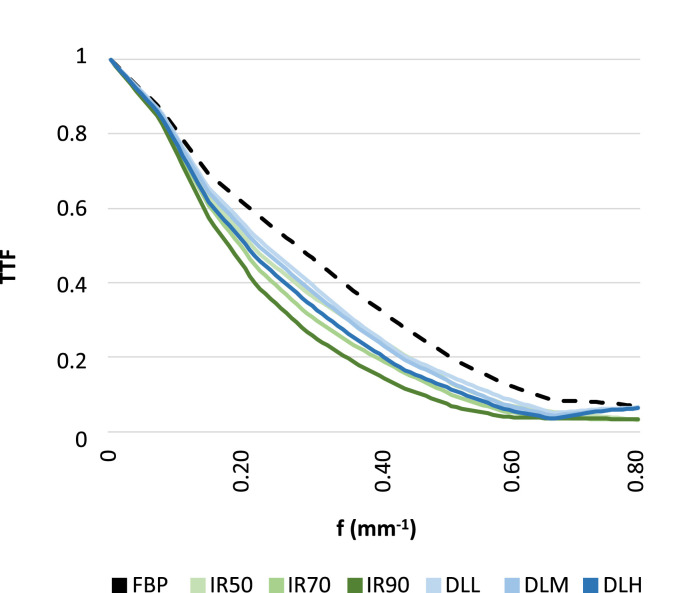
Plot of task-based transfer function (TTF) for a low contrast feature at the 25 mGy dose level for images reconstructed with filtered back projection (FBP), three levels of hybrid iterative reconstruction (IR50, IR70 and IR90) and three strengths of deep learning reconstruction (low [DLL], medium [DLM] and high strength [DLH]) in 2.5 mm slice thickness.

TTF curves for the low-contrast insert for each reconstruction algorithm are presented in Fig. 5. For the 25 mGy dose level, sharpness of the low-contrast object was superior for FBP images compared to both IR and DLR for the same dose level, whereas DLR was superior to IR where f_50%_ values for 2.5 mm images were 24%, 30% and 37% lower than FBP for IR50, IR70 and IR90, respectively, whereas f_50%_ values were 16%, 20% and 27% lower for DLL, DLM and DLH, respectively.

**Fig. 6:**
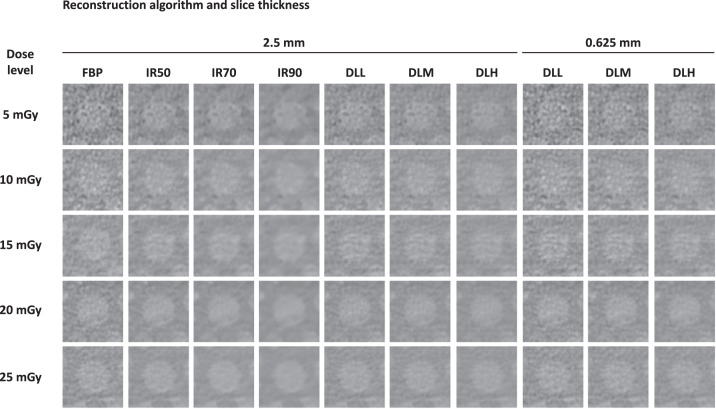
Selection of CT images exhibiting the phantom low contrast lesion, reconstructed with filtered back projection (FBP), three levels of hybrid iterative reconstruction (IR50, IR70 and IR90) and three strengths of deep learning reconstruction (low [DLL], medium [DLM] and high strength [DLH]) for each investigated dose level.

A visual comparison of the low-contrast lesion for each dose level, reconstruction algorithm, and slice thickness is presented in Fig. 6. Careful visual inspection comparing increasing strengths of DLR to increasing levels of hybrid IR showed a perceivable difference in lesion edge sharpness in favor of the DLR images, despite comparable levels of noise reduction.

## 4 Discussion

This study demonstrates that CT image noise is markedly reduced when applying a DLR algorithm. Contrary to increasing levels of hybrid IR, noise reduction with higher strengths of DLR is achieved without compromising noise texture characteristics. Furthermore, images reconstructed with DLR in thin 0.625 mm slices show similar image noise characteristics to 2.5 mm FBP and 2.5 mm IR50, depending on DLR algorithm strength.

Sufficient image quality to detect sometimes subtle anatomical changes indicative of disease is essential in medical imaging. Traditionally, obtaining higher quality images has required higher radiation dose delivered to the patient, with associated increased long-term radiation-related risks [20]. Whereas FBP has been the historical gold standard for CT image reconstruction since the clinical introduction of CT scanners, several IR algorithms have emerged with demonstrated potential for dose reduction [6, 21]. However, application of IR algorithms not only affects image noise, but also alters image texture. Concerns have been raised this may deteriorate image quality, where CT images may appear diagnostically acceptable but fail to show important clinical information – particularly when radiation dose is reduced below a certain threshold [8]. Studies have demonstrated that low-contrast detectability is preserved for only modest levels of dose reduction with IR, up to approximately 25% [22], [23], [24], [25]. Thus, novel methods to reduce image noise are aspired in clinical practice in pursuit of dose reduction.

As reviewed by Mileto et al. [8], it is imperative that novel noise reduction methods are comprehensively evaluated in phantom and clinical studies to ensure that established clinical benefits of CT are not compromised, such as precise and consistent HU measurements. This study confirms high degree of CT number consistency across reconstruction algorithms and dose levels, with average CT number in the homogenous insert differing only 5.8 HU between CT images and homogeneity (i.e., the largest difference in mean CT number within the ROIs in the homogenous insert) up to 4.6 HU for the images reconstructed with 2.5 mm DLR, and 8.2 HU for 0.625 mm DLR. This is arguably within expected standards of a CT system, exemplified by requirements from the International Electrotechnical Commission of uniformity within +/- 4 HU of nominal value, e.g., in a water phantom [26].

Furthermore, this study demonstrates that the DLR algorithm markedly reduces CT image noise at levels comparable to hybrid IR, with noise reduction achieved with DLL, DLM and DLH roughly paralleling that of IR50, IR70 and IR90, respectively. Noticeably, this noise reduction is achieved without altering noise texture, where NPS curves for images reconstructed with DLR paralleled that of FBP, whereas NPS f_avg_ was progressively lower than for FBP with higher levels of IR. The robust denoising property of the DLR algorithm may indicate significant potential for dose reduction, which may be especially attractive in a screening setting, pediatric imaging, and for patients in the need of repeated CT examinations. However, additional studies in a clinical setting incorporating low-contrast tasks and diagnostic performance across pathologies are required to establish the true dose reduction potential for the DLR algorithm.

Compared to traditional FBP, images with IR are frequently characterized as smooth, blotchy, plastic- or synthetic-looking [27, 28]. These artifacts may potentially hamper interpretation of imaging findings, limiting the full potential for dose-reduction with IR [10]. As explored by Morsbach et al. [7], image noise texture deviation, NTD, is an objective metric for quantifying such IR-specific artifacts, with demonstrated correlation to subjective IR-specific artifacts. Interestingly, this study suggests that the DLR algorithm may have overcome this limitation of IR, exemplified by images reconstructed with DLR demonstrating comparable levels of NTD to FBP contrary to hybrid IR where NTD increased with higher levels of hybrid IR blend. This is also confirmed by careful visual inspection of the lesion-to-background interface across reconstruction algorithms, where images reconstructed with higher levels of IR qualitatively appear more blotchy compared to DLR, and the low contrast lesion edge more blurred (Fig. 6).

When comparing DLR in thin 0.625 mm slices to FBP and IR in thicker 2.5 mm slices, DLL was found to have similar noise magnitude across dose levels to 2.5 mm FBP, and DLH to 2.5 mm IR50. This suggests that reconstructing in thinner slices with DLR has the potential to enhance spatial resolution without compromising perceived image quality, potentially negating the partial volume effect associated with reconstruction in thicker slices. This may be especially beneficial in detection and characterization of smaller objects, being more susceptible to partial volume averaging when reconstructing with thicker slices. Previous studies evaluating liver-CT reconstructed with FBP in thinner slices have found that diagnostic performance for detecting low-contrast lesions is reduced, attributed to an increase in image noise [29]. This limitation of thin slice reconstruction may be overcome by novel de-noising methods, and should be further explored in clinical studies. Additionally, reconstruction with isotropic 0.625 mm voxels has the benefit of more readily being able to visualize structures by use of 3D models or out-of-plane reformatting. Finally, the findings of preserved image quality when reconstructing images in 0.625 mm slices with DLR to 2.5 mm FBP/IR may suggest that significant dose-reduction can be feasible when DLR is reconstructed in 2.5 mm slices.

Several studies have explored the image quality characteristics of this vendor-specific DLR technique. Phantom studies have demonstrated strong noise magnitude reduction compared to FBP while maintaining similar noise texture [[11], [12], [30], [31], [32]]. The current study confirms these findings in a controlled semi-anthropomorphic phantom setting, and in addition demonstrates how thin 0.625 slices compare with conventional 2.5 mm slices across dose levels. Noticeably, although dose reduction potential solely based on image noise measurements seems significant, task-based models have estimated the dose reduction potential to be somewhat lower, and approximately 50% with DLH [11].

Furthermore, a human observer study in a controlled phantom setting found that low-contrast detectability was improved for higher strength of DLR compared to both FBP and IR50, and estimated the dose-reduction potential of DLH relative to IR50 to approximately 40% [33]. However, a recent study in a clinical setting found that although DLR improved perceived CT image quality compared to FBP, characterization of liver lesions and reader confidence with reduced-dose DLR was inferior compared to standard-dose FBP [14]. Thus, how noise reduction using DLR translates to dose reduction in the clinics remains unknown.

This study is not without limitations. First, as the DLR algorithm is designed to process complex patient images, a phantom study may not fully demonstrate the full potential of the novel reconstruction technique. Second, in this semi-anthropomorphic phantom approach, the homogeneous insert applied in the study was relatively small limiting ROI size for the NPS measurements. Furthermore, the semi-anthropomorphic design may be a cause of noise non-uniformity affecting the NPS measurements. In this regard, it is important to note that our NPS findings coincide with other studies performed on tailored phantoms only to validate this in a semi-anthropomorphic setting [11]. Third, the low-contrast insert applied is not tailored for TTF analyses where ROI size radius had to be decreased to slightly less than twice of rod radius to avoid neighboring structures. Furthermore, contrast-to-noise conditions were suboptimal, also for the highest dose levels. Thus, these results should be interpreted with care although our findings are in line with other studies performed on low-contrast lesions under more favorable conditions [12]. Nevertheless, studies investigating sharpness of low-contrast features and lesion detectability in a clinical setting are aspired to characterize the clinical benefit of the DLR algorithm. Finally, as this study applied standard parameters and kernels for abdominal imaging, it is unknown how these findings translate to other body regions, for example when it comes to detection of subtle features in cerebrovascular imaging or pulmonary nodule assessment in thoracic imaging.

In conclusion, this study demonstrates that CT images reconstructed with a DLR algorithm shows improved quantitative image quality characteristics when compared to traditional FBP and IR. Demonstrating robust noise reduction while maintaining noise texture characteristics, this has promising potential for dose reduction. Furthermore, there may be additional benefit from image reconstruction in thin slices. Additional studies incorporating low-contrast tasks and diagnostic performance across pathologies are required to further characterize the clinical benefit of the DLR algorithm.

## Ethical approval

Ethical approval was not required in this phantom-only study.

## Funding sources

This work did not receive any specific funding.

## CRediT authorship contribution statement

**Tormund Njølstad:** Conceptualization, Methodology, Investigation, Writing – original draft. **Anselm Schulz:** Conceptualization, Writing – review & editing, Supervision. **Kristin Jensen:** Data curation, Investigation, Writing – review & editing. **Hilde K. Andersen:** Investigation, Supervision, Writing – review & editing. **Anne Catrine T. Martinsen:** Investigation, Supervision, Writing – review & editing, Project administration.

## Declaration of Competing Interest

This study is part of ongoing research at the Oslo University Hospital CT Research and Technology Group. Oslo University Hospital has institutional research agreements with GE Healthcare and The Phantom Laboratory, among others. The authors of this article had complete control of data that might have presented a conflict of interest throughout the study period, and the decision to publish has been at the sole discretion of the authors. The authors state no individual conflicts of interests.

## Appendix Supplementary materials

Image, application 1
