# Supplementary figures and images for: Improved image quality with deep learning reconstruction – a study on a semi-anthropomorphic upper-abdomen phantom
Source: Res Diagn Interv Imaging. 2023 Jan 13;5:100022. doi: 10.1016/j.redii.2023.100022 (PMC11265485; doi:10.1016/j.redii.2023.100022)

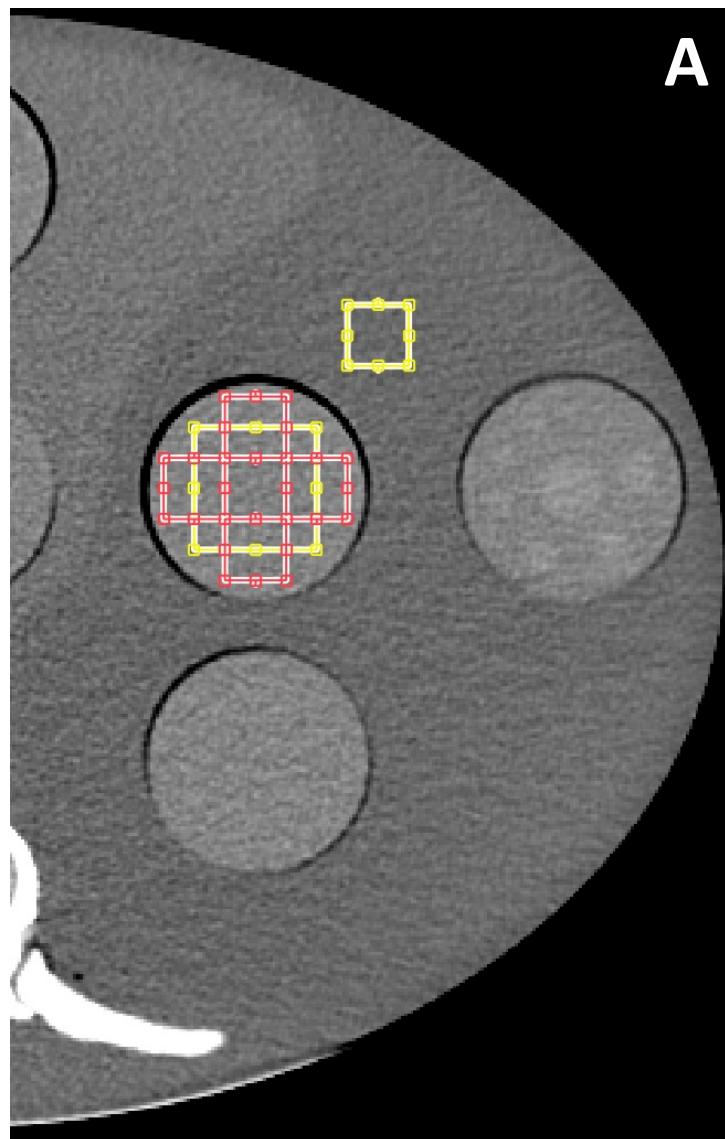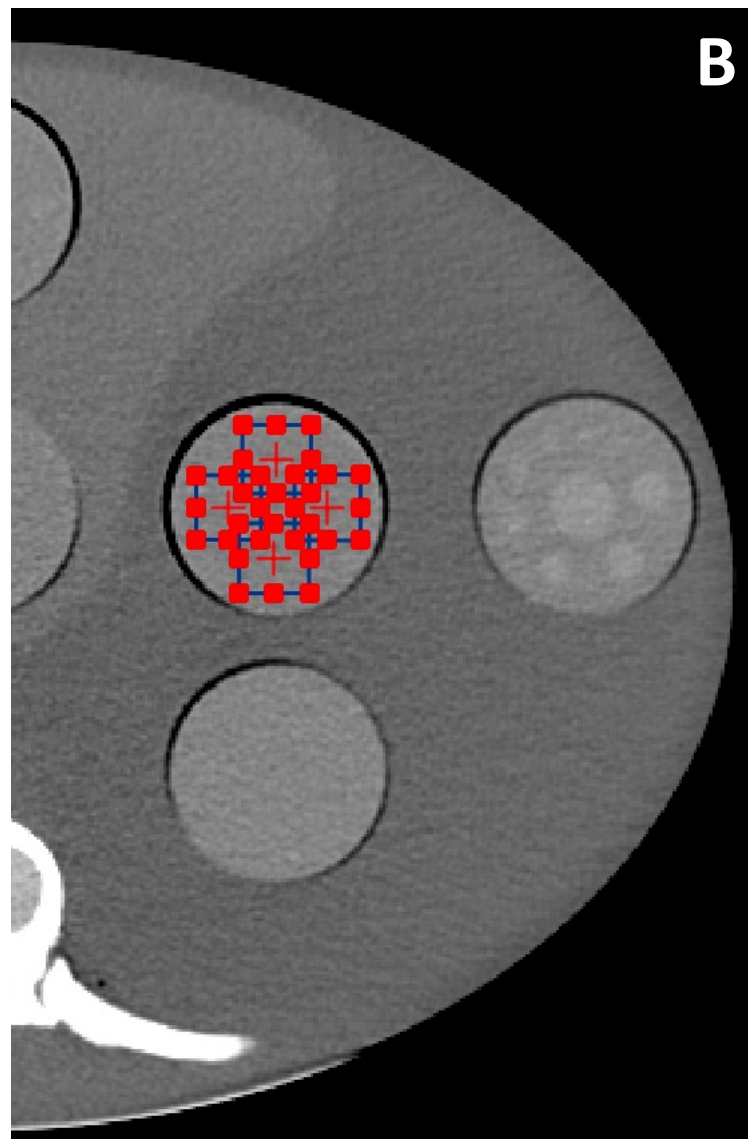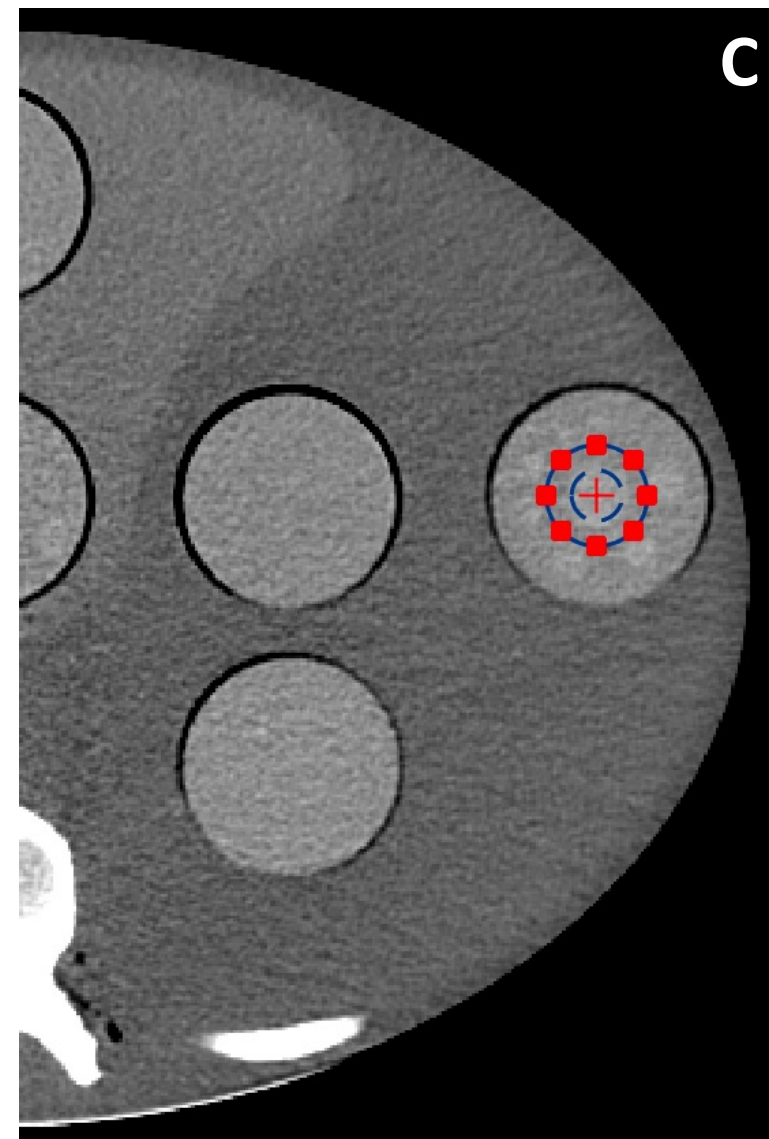

Supplement: Supplementary file 1 [file mmc1.pdf]
